# Supplementary material for: KLF10 integrates circadian timing and sugar signaling to coordinate hepatic metabolism
Source: eLife. 2021 Aug 17;10:e65574. doi: 10.7554/eLife.65574 (PMC8410083; doi:10.7554/eLife.65574)
Supplement: Transparent reporting form [file elife-65574-transrepform1.docx]

***eLife’s* transparent reporting form**

We encourage authors to provide detailed information *within their submission* to facilitate the interpretation and replication of experiments. Authors can upload supporting documentation to indicate the use of appropriate reporting guidelines for health-related research (see [EQUATOR Network](http://www.equator-network.org/%20)), life science research (see the [BioSharing Information Resource](https://biosharing.org/)), or the [ARRIVE guidelines](http://www.plosbiology.org/article/info:doi/10.1371/journal.pbio.1000412) for reporting work involving animal research. Where applicable, authors should refer to any relevant reporting standards documents in this form.

If you have any questions, please consult our Journal Policies and/or contact us: [editorial@elifesciences.org](mailto:editorial@elifesciences.org).

**Sample-size estimation**

- You should state whether an appropriate sample size was computed when the study was being designed
- You should state the statistical method of sample size computation and any required assumptions
- If no explicit power analysis was used, you should describe how you decided what sample (replicate) size (number) to use

Please outline where this information can be found within the submission (e.g., sections or figure legends), or explain why this information doesn’t apply to your submission:

No power analysis was performed prior to the study experiments. Sample size was empirically determined based on both earlier similar measurements done in our lab (eg. gene expression and tolerance tests) and pilot experiments.

**Replicates**

- You should report how often each experiment was performed
- You should include a definition of biological versus technical replication
- The data obtained should be provided and sufficient information should be provided to indicate the number of independent biological and/or technical replicates
- If you encountered any outliers, you should describe how these were handled
- Criteria for exclusion/inclusion of data should be clearly stated
- High-throughput sequence data should be uploaded before submission, with a private link for reviewers provided (these are available from both GEO and ArrayExpress)

Please outline where this information can be found within the submission (e.g., sections or figure legends), or explain why this information doesn’t apply to your submission:

Each experiment was performed using 3 to 12 biological replicates depending of the type of experiment when using animals or primary cells. The number of biological replicates is indicated in all figure legends. For the circadian gene expression, pools of 3 livers were used as indicated in the method section.

We have used weight gain in the high sugar experiment as an inclusion criteria (see line 442 in the method section).

In the absence of objective criteria (eg. value out of standard curve range, stressed animal) outliers were kept as non-parametric testing based on ranks does not overestimate the weight of outliers.

RNA-seq data and Chip-seq data have been deposited at European Nucleotide Archive as indicated in the Data availability section.

**Statistical reporting**

- Statistical analysis methods should be described and justified
- Raw data should be presented in figures whenever informative to do so (typically when N per group is less than 10)
- For each experiment, you should identify the statistical tests used, exact values of N, definitions of center, methods of multiple test correction, and dispersion and precision measures (e.g., mean, median, SD, SEM, confidence intervals; and, for the major substantive results, a measure of effect size (e.g., Pearson's r, Cohen's d)
- Report exact p-values wherever possible alongside the summary statistics and 95% confidence intervals. These should be reported for all key questions and not only when the p-value is less than 0.05.

Please outline where this information can be found within the submission (e.g., sections or figure legends), or explain why this information doesn’t apply to your submission:

Because the normal distribution cannot be tested accurately with small samples, we have used the Wilicoxon and Kruskal-Wallis non parametric tests for pairwise or multiple comparisons respectively. Kruskal-Wallis testing was further adjusted using the Benjamini-Hochberg post-hoc test to account for multiple testing. Similarly, the MetaCycle method used to detect circadian oscillations is based on non-parametric testing. A three-way ANOVA was performed for the GTT experiment reported in figure 4. Data are presented as mean ± SEM. This information is provided in all figures when applicable. Exact p-values can be found in the source data files and supplemental tables.

(For large datasets, or papers with a very large number of statistical tests, you may upload a single table file with tests, Ns, etc., with reference to sections in the manuscript.)

**Group allocation**

- Indicate how samples were allocated into experimental groups (in the case of clinical studies, please specify allocation to treatment method); if randomization was used, please also state if restricted randomization was applied
- Indicate if masking was used during group allocation, data collection and/or data analysis

Please outline where this information can be found within the submission (e.g., sections or figure legends), or explain why this information doesn’t apply to your submission:

For animal experiments testing the effect of high sugar, animals were randomly allocated to each experimental group as indicated in the method section. Non restricted randomization was used. Analysis of steatosis on tissue section was done blind as indicated in the method section.

**Additional data files (“source data”)**

- We encourage you to upload relevant additional data files, such as numerical data that are represented as a graph in a figure, or as a summary table
- Where provided, these should be in the most useful format, and they can be uploaded as “Source data” files linked to a main figure or table
- Include model definition files including the full list of parameters used
- Include code used for data analysis (e.g., R, MatLab)
- Avoid stating that data files are “available upon request”

Please indicate the figures or tables for which source data files have been provided:

Source data are provided as excel files for Figures 1-7. Sequencing data related to Figures 2, 6 and 7 have been deposited at ENA.
